# Supplementary material for: Identification of photoperiod-induced specific miRNAs in the adrenal glands of Sunite sheep (Ovis aries)
Source: Front Vet Sci. 2022 Jul 22;9:888207. doi: 10.3389/fvets.2022.888207 (PMC9354845; doi:10.3389/fvets.2022.888207)
Supplement: Supplementary file 1 [file Data_Sheet_1.ZIP › Supplementary file/miRDeep2 file.zip!/miRDeep2 file/Novel_Structure/SP42A2/Novel_621.pdf]

Provisional ID	:	NC_019471.2_27792
Score total	:	122.4
Score for star read(s)	:	3.9
Score for read counts	:	114.3
Score for mfe	:	2.6
Score for randfold	:	1.6
Score for cons. seed	:	
Total read count	:	236
Mature read count	:	230
Loop read count	:	0
Star read count	:	6

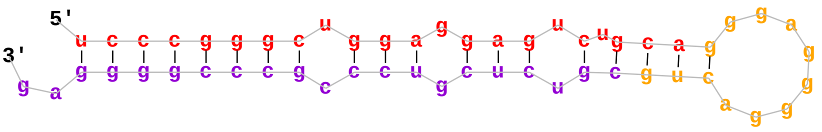

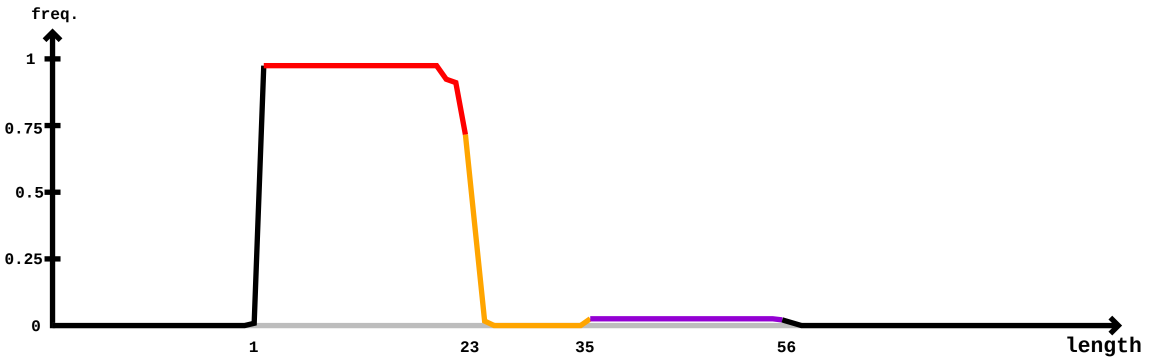

Mature Star

```
5'- ggaccugggcaggggcggggucccgggcuggaggagucugcagggaggggacugcgucucgucccgcccggggagaccgccccuugccgcucccaggacgguggauagc  -3'   obs
    ggaccugggcaggggcggggucccgggcuggaggagucugcagggaggggacugcgucucgucccgcccggggagaccgccccuugccgcucccaggacgguggauagc         exp
    .......(((((((((((..((((((((.(((.(((.(.((((........))))).))).))).))))))))...))))))).))))(((.(((......)))..)))     reads    mm     sample
    ...................gucccgggcuggaggagucugc....................................................................     1        0      seq
    ...................gucccgggcuggaggagucugca...................................................................     1        0      seq
    ....................ucccgggcuggaggagucu......................................................................     12       0      seq
    ....................ucccgggcuggaggagucug.....................................................................     3        0      seq
    ....................ucccgggcuggaggagucugc....................................................................     45       0      seq
    ....................ucccgggcuggaggagucugca...................................................................     103      0      seq
    ....................ucccgggcuggaggagucugcag..................................................................     61       0      seq
    ....................ucccgggcuggaggagucugcagg.................................................................     4        0      seq
    ......................................................cgucucgucccgcccgggga...................................     1        0      seq
    ......................................................cgucucgucccgcccggggag..................................     5        0      seq
```
